# Survival of epithelial ovarian cancer in Black women: a society to cell approach in the African American cancer epidemiology study (AACES)

**DOI:** 10.1007/s10552-022-01660-0

**Published:** 2022-12-15

**Authors:** Joellen M. Schildkraut, Courtney Johnson, Lauren F. Dempsey, Bo Qin, Paul Terry, Maxwell Akonde, Edward S. Peters, Hannah Mandle, Michele L. Cote, Lauren Peres, Patricia Moorman, Ann G. Schwartz, Michael Epstein, Jeffrey Marks, Melissa Bondy, Andrew B. Lawson, Anthony J. Alberg, Elisa V. Bandera

**Affiliations:** 1 Department of Epidemiology, Rollins School of Public Health, Emory University, Atlanta, GA USA; 2 Cancer Epidemiology and Health Outcomes, Rutgers Cancer Institute of New Jersey, New Brunswick, NJ USA; 3 Department of Medicine, University of Tennessee Medical Center-Knoxville, Knoxville, TN USA; 4 Department of Epidemiology and Biostatistics, Arnold School of Public Health, University of South Carolina, Columbia, SC USA; 5 Department of Epidemiology, College of Public Health, University of Nebraska Medical Center, Omaha, NE USA; 6 Indiana University Melvin and Bren Simon Comprehensive Cancer Center, Indiana University, Indianapolis, IN USA; 7 Department of Cancer Epidemiology, H. Lee Moffit Cancer Center and Research Institute, Tampa, FL USA; 8 Department of Family Medicine and Community Health, Duke University School of Medicine, Durham, NC USA; 9 Department of Oncology, Karmanos Cancer Institute, Wayne State University School of Medicine, Detroit, MI USA; 10 Department of Surgery, Duke University School of Medicine, Durham, NC USA; 11 Department of Epidemiology and Population Health, Stanford University School of Medicine, Stanford, CA USA; 12 Department of Public Health Sciences, College of Medicine, Medical University of South Carolina, Charleston, SC USA

**Keywords:** Epithelial ovarian cancer, Cancer survivors, Racial disparities, Social determinants of health

## Abstract

**Purpose:**

The causes for the survival disparity among Black women with epithelial ovarian cancer (EOC) are likely multi-factorial. Here we describe the African American Cancer Epidemiology Study (AACES), the largest cohort of Black women with EOC.

**Methods:**

AACES phase 2 (enrolled 2020 onward) is a multi-site, population-based study focused on overall survival (OS) of EOC. Rapid case ascertainment is used in ongoing patient recruitment in eight U.S. states, both northern and southern. Data collection is composed of a survey, biospecimens, and medical record abstraction. Results characterizing the survival experience of the phase 1 study population (enrolled 2010–2015) are presented.

**Results:**

Thus far, ~ 650 patients with EOC have been enrolled in the AACES. The five-year OS of AACES participants approximates those of Black women in the Surveillance Epidemiology and End Results (SEER) registry who survive at least 10-month past diagnosis and is worse compared to white women in SEER, 49 vs. 60%, respectively. A high proportion of women in AACES have low levels of household income (45% < $25,000 annually), education (51% ≤ high school education), and insurance coverage (32% uninsured or Medicaid). Those followed annually differ from those without follow-up with higher levels of localized disease (28 vs 24%) and higher levels of optimal debulking status (73 vs 67%).

**Conclusion:**

AACES is well positioned to evaluate the contribution of social determinants of health to the poor survival of Black women with EOC and advance understanding of the multi-factorial causes of the ovarian cancer survival disparity in Black women.

**Supplementary Information:**

The online version contains supplementary material available at 10.1007/s10552-022-01660-0.

## Introduction

The five-year relative survival for ovarian cancer is worse for Black women, at 41%, when compared with White women, at 48% [1]. After stratifying on stage at diagnosis, survival is worse in Black women compared to any other racial group for both early- and late-stage disease [2]. Factors affecting epithelial ovarian cancer (EOC) prognosis are under-studied among Black women despite notable differences in age at diagnosis, clinical-pathological features, and survival compared to White women [3]. The causes for this survival disparity are most likely multi-factorial.

Social determinants of health could contribute to poorer survival in Black women. Lower socioeconomic status (SES), with limited access to care, may lead to more advanced stage at diagnosis and receipt of suboptimal treatment [4]. Social determinants of health can also contribute to individual-level susceptibility factors that lead to poorer prognosis, such as higher prevalence of comorbidities and a higher inflammatory response [5, 6]. A pooled analysis from the Ovarian Cancer in Women of African Ancestry (OCWAA) consortium suggested that several factors including reproductive factors, comorbid conditions, and hormone use appear to mediate ovarian cancer survival differences between Black and White women [7].

**Fig. 1:**
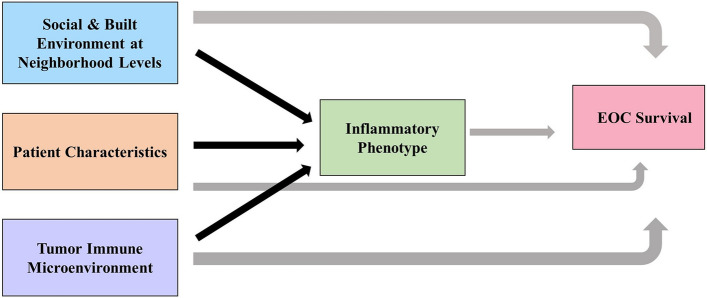
Conceptual model demonstrating the multi-level constructs examined in relationship to ovarian cancer survival

To date, no cohort study has focused on Black EOC survivors. Here we present the African American Cancer Epidemiology Study (AACES) [8], the largest cohort of Black women with EOC residing in several U.S. states where we will further explore why EOC survival is poor among Black women. We are pursuing a society to cell approach (Fig. 1), facilitated through multi-level modeling, to address effects at different levels: social determinants of health at the neighborhood and census tract levels that may contribute to disparities in ovarian cancer survival (i.e., deprivation and segregation indices); individual-level factors such as inflammatory-related lifestyle exposures factors, factors known to be associated with cancer survival (e.g., cigarette smoking), and social determinants of health such as socioeconomic status and measures of structural racism (i.e., trust in physicians and perceived discrimination); and tumor (e.g., stage) and cellular features (i.e., tumor immune microenvironment). In AACES we focus on inflammatory factors, given the strong role of inflammation in ovarian carcinogenesis and growing evidence of its role in prognosis [5].

Given its size, geographical diversity, and breadth of data, including biospecimens, the AACES cohort provides a unique resource to advance understanding of the multi-factorial causes of the ovarian cancer survival disparity in Black women. The intent of this paper is to provide a detailed roadmap to our approach in our ongoing, multi-site study of Black women diagnosed with EOC, a rare and highly fatal disease. After assessing risk factor associations in phase 1, our current study goals in phase 2 are focused on predictors of overall survival pursuing a multi-level society to cell approach to address effects at different levels. The role of the analysis in the current manuscript is descriptive, while future analyses will examine predictors of overall survival using a multi-level approach. For this reason, the methods section provides detailed information about the study design and conduct.

## Methods

### Study population

Phase 1 of the AACES was initiated in 2010 as a multi-site population-based, case–control study, described in detail elsewhere [8]. The study was designed to evaluate genetic and lifestyle risk factors for EOC in Black women, with the intention of also conducting follow-up of the participants to evaluate survival in future. Phase 2 of AACES, which began in 2020, builds upon the infrastructure developed in the AACES phase 1 and focuses on evaluating associations of multi-level factors (neighborhood, lifestyle, and biological factors) with overall survival (OS) of EOC using a prospective cohort design, which combines participants enrolled in AACES phase 1 and newly recruited participants in phase 2. The chosen study sites (Supplemental fig 1) [9] represent geographic diversity as well as an approach for an efficient means for accrual of minority women diagnosed with a rare cancer. Consideration was also given for the proportion of Black residents, the incidence rates of ovarian cancer, and the frequency of newly diagnosed cases of EOC within the geographic regions.

In phase 1, the study sites were selected based on geographic regions with high Black population density and included Alabama, Georgia, Louisiana, metropolitan Detroit, Illinois, New Jersey, North Carolina, Ohio, South Carolina, Tennessee, and Texas. Potential participants were eligible if they had histologically confirmed EOC, self-identified as African American or Black, were 20–79 years of age at diagnosis, and had the ability to complete the interview in English. Rapid case ascertainment was used to identify potential participants with newly diagnosed EOC through state cancer or Surveillance Epidemiology and End Results (SEER) registries and through gynecologic oncology departments at individual hospitals. Participants were recruited between December 2010 and 2015. The data presented in this paper are from women enrolled in phase 1. Based on the AACES phase 1 design and infrastructure, we are currently enrolling women newly diagnosed with invasive epithelial ovarian cancer in phase 2. Our goal is to increase the number of participants recruited in AACES by ~ 50% (*n* = 300 new participants) and focus on factors associated with OS. The data obtained from women enrolled in phase 2 will be used to annotate biospecimen data. Based on the enrollment in phase 1, we chose a subset of the sites for phase 2 with the largest number of enrolled participants, including the states of Georgia, New Jersey, Louisiana, South Carolina, North Carolina, Tennessee, and the Detroit metropolitan area. A new site, the Southern California SEER registry, is also included in phase 2. Four of the sites participating in phase 2, Georgia, New Jersey, Louisiana, and Southern California, are SEER registries. All sites employ rapid case ascertainment, described below. Informed consent was obtained from all individual participants included in the study. We have established a single IRB using the Western Institutional Review Board-Copernicus Group (WCG IRB) that all sites rely on.

**Fig. 2:**
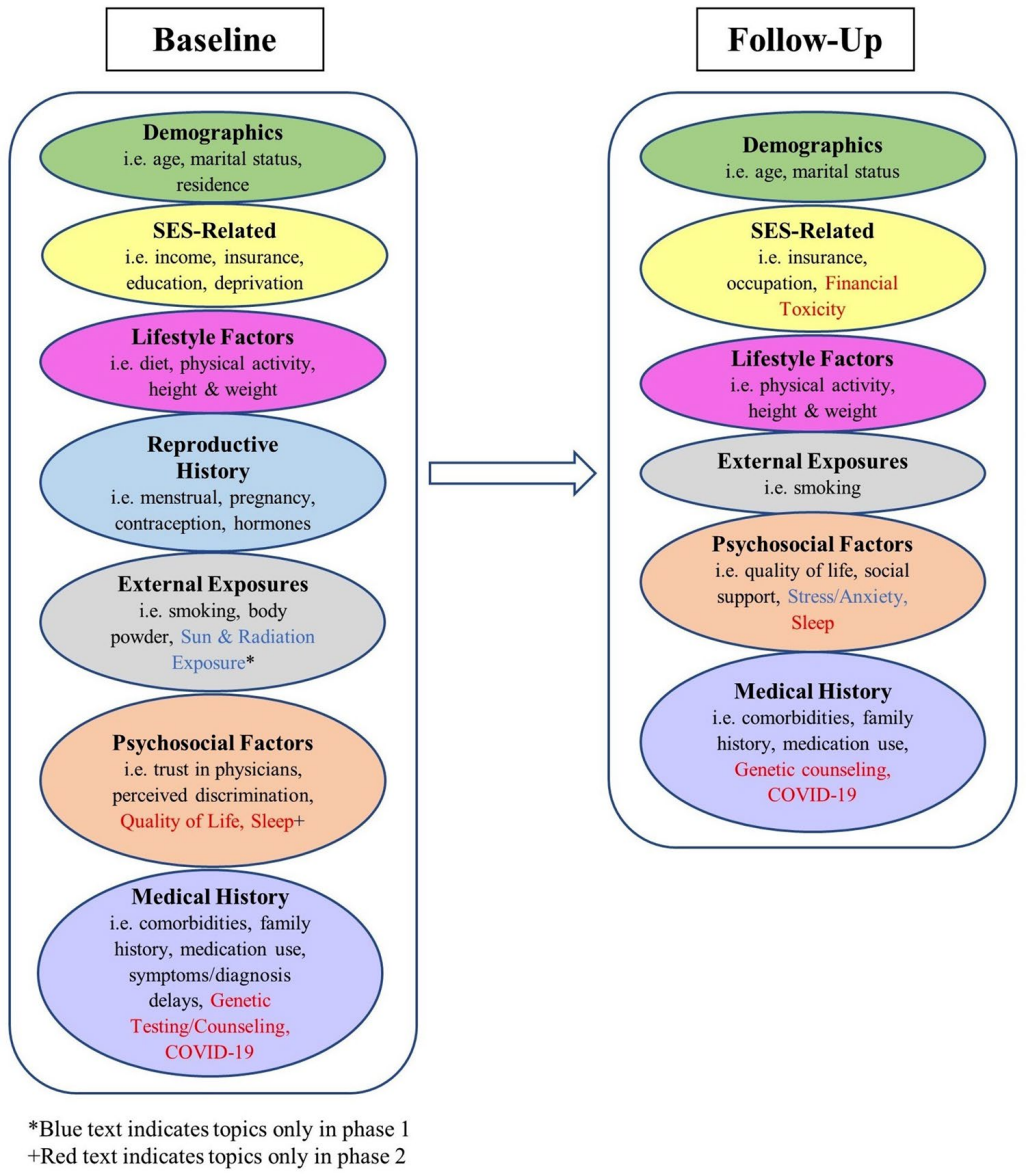
Baseline & Follow-Up Questionnaires

In phase 2, we continue to incorporate the majority of survey questions and indices used in phase 1 to maximize our ability to pool data from the two phases of AACES. However, we have updated some survey questions when we determined improvement was required and added other constructs of interest that were not collected in phase 1, such as financial toxicity (Fig. 2). We now obtain information about genetic counseling in a population that has been shown to be deficient in BRCA1 and BRCA2 testing [10] and we obtain information concerning COVID 19, as phase 2 began during the beginning of the COVID 19 pandemic.

After passive approval from the physician, potential participants from Georgia, New Jersey, and South Carolina are contacted by their cancer registry by mail and by telephone. Participant approval (verbally or by mail) is required from these sites before their contact information is passed to the study team. Participants from California, Louisiana, North Carolina, and Tennessee do not require active approval from the patient to send participant contact information to the study team. Some hospitals in the Detroit metropolitan area require active physician approval before reaching out to the potential participant. This information is displayed in Table 1.

**Table 1:**
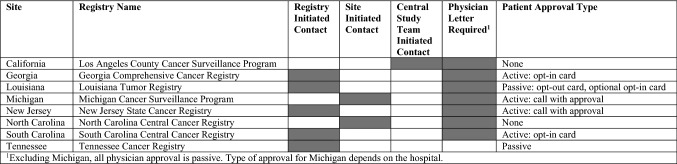
Initial patient identification and process of approval to be contacted by the central study team by site

^1^Excluding Michigan, all physician approval is passive. Type of approval for Michigan depends on the hospital

### Enrollment and retention strategies

All potential study participants are mailed a packet of introductory material. Consent can be obtained verbally for the study survey, and written consent is obtained for other study activities. The study team reaches out to the participant to complete the food frequency questionnaires, the annual follow-up surveys, and making medical records requests and requests for paraffin-embedded tumor tissue samples. To mitigate obstacles when contacting participants, we implemented several strategies including an identifiable study logo. Additionally, a brochure that highlights aspects of study participation and compensations is mailed to each potential study participant. We also provide a website (https://aaces.emory.edu/) and a toll-free number to accommodate those who want more information. An informational one-page flyer is sent prior to initiating phone contact to alert the patient about the study, informing the patient that she will be contacted by phone. All telephone numbers that are involved with patient contact have a caller ID labeled as “AACES Study Office” or “AACES Study.”

Multiple call attempts are made to reach the potential participant at different times of the day including early evening and weekends. Study participants are given alternative options to complete the survey online or on a paper version if the baseline telephone interview does not offer a desired time commitment and flexibility. Newsletters are sent twice a year that includes recent publications, study updates, and educational materials on topics related to cancer survivorship. The newsletters help to maintain up-to-date contact information. A $50.00 compensation is paid to participants who complete the baseline survey.

#### Baseline and follow-up surveys

In phase 1, participants completed a baseline telephone survey that collected information on sociodemographic characteristics, medical history, self-reported family history of cancer, lifestyle characteristics [11], perceived daily and lifetime discrimination [12], social support [13, 14], religiosity [15], health insurance status, health care provider access, and trust in physicians [16]. For phase 2, some survey content was revised: social support was switched from the Multidimensional Scale of Perceived Social Support (MSPSS) [13] to the modified Medical Outcomes Study Social Support Survey (mMOS-SS) [14]. We also added additional questions to the phase 2 baseline survey including the Short Form Health Survey (SF-12 version 2) quality of life [17], the Pittsburgh sleep quality index [18], an index for financial toxicity [19], COVID 19 diagnosis, and vaccination, and questions pertaining to genetic testing and counseling (Fig. 2). Both phase 1 and phase 2 include a follow-up survey at approximately one year after baseline interview that addresses changes and updates such as changes in physical activity [11], employment, quality of life (SF-8 in phase 1 and SF-12 version 2 in phase 2) [17, 20], medication use, new diagnoses of cancer and other comorbid conditions in the participant and family members, and genetic counseling and testing. In phase 2, study data for baseline and follow-up surveys were collected and managed using Research Electronic Data Capture (REDCap) hosted at Emory University [21, 22]. The online and hardcopy version of the baseline survey has an abbreviated assessment of some factors including parity, oral contraceptive use, family history, access to care, and symptoms and provides flexibility for the participant who requests this option.

#### Geocoding and area-level variables

In order to link neighborhood-level variables, we use the participant’s residential address at diagnosis and geocode to latitude and longitude coordinates. These data can be linked to various levels of geospatial data related to indices of socioeconomic status and/or deprivation, environmental pollution, measures of access to care, and more [23–28]. This will enable a wider variety and scope of analyses in future using the AACES sample population.

#### Biospecimen collection

Upon receipt of a written informed consent, we initiate collection of biospecimens, including blood or saliva and tumor tissue. When blood or saliva is obtained, a second $50.00 compensation is paid. In both phases of AACES, we contracted with an outside vendor for blood or saliva collection and anthropometric measurements. If a participant is unwilling or contraindicated to give a blood sample, we offer the option to provide a saliva sample.

For participants who signed a specimen release form, study staff contacts the pathology department at the institution where the patient was diagnosed and request pathology reports and formalin-fixed paraffin-embedded (FFPE) tumor blocks that are representative of the primary ovarian cancer. Emory prepares 25 5-micron slides and selects three cores from the primary tissue to assemble tissue microarrays (TMAs). A pathology slide is prepared for each of the tissues received and stained with hematoxylin and eosin (H&E). Immunofluorescent staining of selected immune markers is in process for both phases [29]. We genotyped all study subjects enrolled in Phase 1 with an available germline DNA sample using the Illumina OncoArray [30]. In phase 2, we are considering using an array that better addresses genetic variation in minority populations [31].

#### Pathology criteria

Eligible participants for phase 2 were based on International Classification of Diseases for Oncology, 3rd Edition (ICD-O-3) site and morphology codes for EOC which incorporate 2021 updates in the WHO *Classification of Tumors*, 5th Edition, Volume 4: Female Genital Tumors (see Supplemental table 1). The eligible ICD-O cancer sites are ovary (C56.9), fallopian tube (C57.0), retroperitoneum (C48.0), specified parts of the peritoneum (C48.1), peritoneum (C48.2), overlapping lesions of retroperitoneum & peritoneum (C48.8), and overlapping lesions of female genital organs: tubo-ovarian (C57.8).

#### Medical record abstraction (acquisition and review)

All study participants in phases 1 and 2, diagnosed with EOC, are asked to sign a medical record release form and tumor tissue release form as well as the name of the institution where the diagnosis took place. Information on each participant’s frontline chemotherapy regimen (neoadjuvant and adjuvant, start and end date, name of agent, number of cycles, dose), debulking surgery (type, date), residual disease, debulking status, CA125 levels before and after adjuvant chemotherapy, white blood cell counts before surgery and treatment, and anthropometric measurements at initiation of chemotherapy is collected to the extent possible.

#### Follow-up and vital status collection

In phase 1 annual follow-up interviews were attempted to collect the information described above, as well as to capture any updates in contact information and length of OS. The goal for time between baseline interview and follow-up was one year. When follow-up interviews were not possible, a thorough search with the National Death Index was implemented to obtain date and cause of death where applicable. We are also in the process of using LexisNexis to further identify vital status and OS. We plan to continue these processes for phase 2.

#### Statistical analysis

##### Variable distribution

We generate descriptive statistics of our study population including basic demographics, clinical characteristics, and inflammatory-related factors that are displayed for all participants at baseline and by follow-up status, which was defined as completion of at least one follow-up survey or not. Demographics include variables, such as age at diagnosis, marital status (single/never married, married/living as married, divorced/separated, widowed), highest level of education achieved (high school or less, some college, college graduate, graduate/professional school), insurance status (uninsured, any Medicaid coverage, Medicare only, combination of private insurance & Medicare, private insurance, other), and total annual family income (< $10,000, $10,000–$24,999, $25,000–$49,999, $50,000–$74,999, $75,000–$100,000, > $100,000). Clinical characteristics included summary stage, FIGO stage, histotype, time between baseline and follow-up surveys (where available), residual disease (residual tumor diameter after cytoreductive debulking surgery; no residual disease, < 1 cm residual tumor diameter, ≥ 1 cm residual tumor diameter, residual disease but unknown size of tumor), and debulking status (optimal [no residual disease or < 1 cm residual tumor diameter], suboptimal [≥ 1 cm residual tumor diameter]); described in further detail below). Physical inactivity is classified into categories of < 2 h per week vs ≥ 2 h per week. Physical Activity Guidelines for Americans (PAGA) consider insufficient physical activity to be < 2.5 h of moderate activity or < 1.25 h of vigorous physical activity [32]. Other factors include aspirin and other non-steroidal anti-inflammatory drug (NSAID) use, body mass index (BMI, kg/m^2^), smoking status (never, former, current), talc use (ever vs never), oral contraceptive duration (never, < 5 years, ≥ 5 years), hormone replacement therapy duration (never, < 5 years, ≥ 5 years), parity (number of full-term pregnancies), prior diagnosis of breast cancer, prior diagnosis of any cancer, excluding breast cancer, and the Charlson comorbidity index [33]. Chi-squared tests of independence were used to compare the distributions of all variables by follow-up completion status, and the *p*-values are displayed.

##### Survival comparisons of AACES participants to SEER data

To better understand the population that is captured by AACES, we used SEER*Stat [34] to compare OS rates from our data to women diagnosed with ovarian cancer between 2008 and 2013 for sufficient sample size and a comparative length of follow-up time. We standardized Kaplan–Meier estimates from Black women in SEER to the AACES age distribution. After fitting several conditional curves based on surviving past specific time points, we determined the OS proportions for Black women within SEER data conditioning on at least 10 months of OS post-diagnosis to account for the low response rates among the sickest women and those with aggressive disease. Additionally, to assess the racial disparity related to OS in our study population we determined the same set of conditional age-standardized Kaplan–Meier estimates for White women. A log-Rank test is used to compare the conditional age-standardized Kaplan–Meier curves from SEER between Black and White women.

We determined age-standardized Kaplan–Meier estimates for the five major histotypes: high-grade serous, low-grade serous, mucinous, clear cell, and endometrioid. Here we also conditioned on at least 10-month OS time to enable comparison of the overall population of Black women with ovarian cancer and the AACES study participants. We also used a Chi-squared test of independence to formally compare the distribution of histotypes in SEER to the distribution within AACES.

## Results

Of 1,720 potential participants interviewers attempted to contact, 1,199 (70%) were actively reached. Of these, 592 (49%) were interviewed and 388 (32%) actively refused. Using rapid case ascertainment, the average and median time from diagnosis to the baseline survey interview for participants in phase 1 were 7.1 and 5.8 months, respectively. Among the 592 patients in phase 1, 540 agreed to provide their medical record and tissue sample (91%). Of these, 497 medical records were obtained (92%) and 437 FFPE tumor tissue samples (81%) were obtained. Phase 1 participants have been followed annually through 2016 and again in 2021, with 228 (39%) participants who remain alive and 364 (61%) participants deceased as of the latest update in 2021. For all women with EOC, OS ranged from 0.5 years to 10.6 years with a median of 4.8 years. A total of 577 (97%) participants survived at least 10-month past diagnosis. The mean and median time to the first follow-up survey was 1.6 and 1.2 years, respectively. Participants with 2 or more follow-up survey completions (*n* = 104) had an average time to first follow-up of 1.3 years (median: 1.2 years).

The participants who did not complete any follow-up survey (*n* = 294) are further described according to whether they died before being contacted or rather were lost to follow-up. During phase 1, interviewing ended in April 2016 and therefore, 70 (24%) participants who were interviewed at baseline in April 2015 or later were not eligible to complete follow-up surveys until phase 2 had begun but were deceased upon the start of phase 2. An additional 67 (23%) participants who did not complete a follow-up survey died within a year of their baseline interview over the course of phase 1 and 157 (53%) did not complete a follow-up survey due to other reasons (i.e., no answer on the phone, phone number no longer in service, active refusal, passive refusal).

**Fig. 3:**
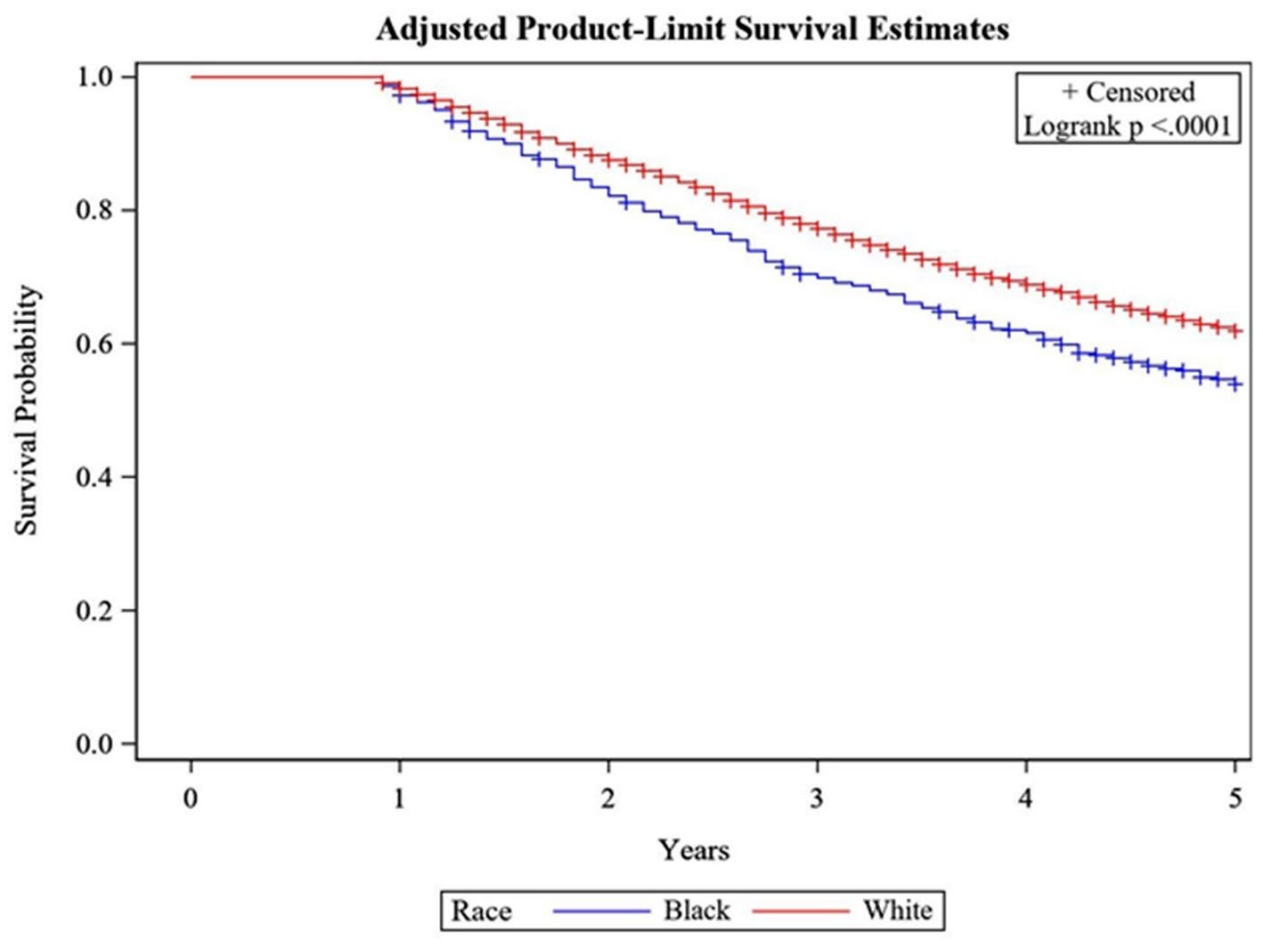
Kaplan–Meier Curves by Race, Age-Standardized to AACES Population, and restricted to survival ≥ 10 months, SEER 2008–2013

As shown in Table 2, AACES phase 1 participants demonstrated better OS than age-standardized Black women in SEER but were virtually the same after conditioning on OS of at least 10 months. Comparing OS of this age-standardized group of Black women in SEER to that of age-standardized White women in SEER (when looking at unrestricted OS and when conditioning on at least a 10-month OS time), Black women have consistently poorer OS (*p*-value < 0.0001) (Fig. 3).

**Table 2:** Overall survival (OS) Rates for AACES and SEER, Black, and White EOC

Months	AACES 2010–2015	SEER AA^a,b^ 2008–2013	SEER AA^a,b^ 2008–2013, given ≥ 10-months survival	SEER White^b,c^ 2008–2013	SEER White^b,c^ 2008–2013, given ≥ 10-month survival
	%, n = 592	%, n= 1,828	%, n = 1,342	%, n = 16,630	%, n = 14,296
12	97	73	97	87	98
24	81	61	80	76	86
36	67	52	67	67	75
48	56	45	58	59	67
60	49	39	51	53	60

Note: 14% (*n* = 2,334) of White women died within 10 months, while 27% (*n*= 486) of Black women died within 10 months

^a^Black women diagnosed with first primary ovarian cancer, restricted to histology codes eligible in AACES 1

^b^Age standardized to AACES age distribution

^c^White women diagnosed with first primary ovarian cancer, restricted to histology codes eligible in AACES 1

### Survey results

Characteristics of the phase 1 participants in AACES are summarized in Table 3. Due to some participants opting to complete an abbreviated version of the survey, variables not included in this version, such as insurance status and annual family income, are missing at higher rates (*n* = 51; 9%). The highest level of education of approximately half of the participants was high school or less (51%), almost half reported a total family income of less than $24,999 annually (45%), one-third of participants were married or living as married (33%), close to one-third of participants were uninsured or had Medicaid coverage (32%), and a further 23% of participants had only Medicare coverage (Table 3). Among women reporting the lowest annual family income in phase 1 (< $10,000), participants were more likely to have Medicaid or no insurance (63%), and have a high school education or less (77%). There were no clear differences in SES-related factors and other basic demographic characteristics by women who completed at least one follow-up survey and those who did not. However, participants who were deceased before the follow-up survey were older at diagnosis than those who could not be followed for other reasons, with a mean age of 61 years (SD = 10.8) compared to 55 years (SD = 11.2) (*p* < 0.001), respectively.

**Table 3:** Characteristics by follow-up completion

	Follow-up status			
	Group 1: full baseline cohort (*n* = 592)	Group 2: completed ≥ 1 follow-up (*n* = 298)	Group 3: no follow-up (*n* = 294)	*p*-value
	*n* (%)	*n* (%)	*n* (%)	
SES-related & other demographic characteristics				
Age at diagnosis (years)				0.45
20–40	29 (4.9)	13 (4.4)	16 (5.4)	
41–60	309 (52.2)	163 (54.7)	146 (49.7)	
61–75	254 (42.9)	122 (40.9)	132 (44.9)	
Education				0.95
High school or less	302 (51.0)	155 (52.0)	147 (50.0)	
Some college	106 (17.9)	51 (17.1)	55 (18.7)	
College graduate	112 (18.9)	56 (18.8)	56 (19.1)	
Graduate/professional school	72 (12.2)	36 (12.1)	36 (12.2)	
Marital status				0.61
Single/never married	142 (24.0)	65 (21.8)	77 (26.2)	
Married/living as married	195 (32.9)	103 (34.6)	92 (31.3)	
Divorced/separated	166 (28.0)	86 (28.9)	80 (27.2)	
Widowed	89 (15.0)	44 (14.8)	45 (15.3)	
Insurance status (before diagnosis)				0.12
Uninsured	50 (9.3)	28 (10.0)	22 (8.5)	
Any medicaid	122 (22.6)	57 (20.3)	65 (25.2)	
Medicare only	123 (22.8)	61 (21.7)	62 (24.0)	
Private & medicare	25 (4.6)	18 (6.4)	7 (2.7)	
Private (only or with other)	189 (35.1)	105 (37.4)	84 (32.6)	
Other	30 (5.6)	12 (4.3)	18 (7.0)	
Unknown	53 (–)	17 (–)	36 (–)	
Total family income (year before diagnosis)				0.39
Less than $10,000	115 (21.7)	52 (18.7)	63 (24.9)	
$10,000 to $24,999	125 (23.5)	72 (25.9)	53 (20.9)	
$25,000 to $49,999	132 (24.9)	74 (26.6)	58 (22.9)	
$50,000 to $74,999	79 (14.9)	39 (14.0)	40 (15.8)	
$75,000 to $100,000	48 (9.0)	26 (9.4)	22 (8.7)	
More than $100,000	32 (6.0)	15 (5.4)	17 (6.7)	
Missing	61 (–)	20 (–)	41 (–)	
Rural–urban commuting area**				0.01
Metropolitan	467 (86.6)	251 (88.4)	216 (84.7)	
Micropolitan	44 (8.2)	14 (4.9)	30 (11.8)	
Small town	18 (3.3)	11 (3.9)	7 (2.7)	
Rural	10 (1.9)	8 (2.8)	2 (0.8)	
Missing	53 (–)	14 (–)	39 (–)	
Inflammation-related factors				
Anti-inflammatory medications before diagnosis				
Aspirin	90 (16.9)	41 (14.6)	49 (19.3)	0.15
other NSAID	117 (21.9)	63 (22.5)	54 (21.3)	0.73
BMI (kg/m^2^—self-reported at initial interview)				0.86
< 18.5	5 (0.9)	2 (0.7)	3 (1.0)	
[18.5, 25)	81 (13.8)	44 (14.8)	37 (12.7)	
[25, 30)	155 (26.4)	78 (26.3)	77 (26.5)	
30 +	347 (59.0)	173 (58.2)	174 (59.8)	
Missing	4 (–)	1 (–)	3 (–)	
Smoking status				0.15
Never	327 (55.2)	176 (59.1)	151 (51.4)	
Former	205 (34.6)	96 (32.2)	109 (37.1)	
Current	60 (10.1)	26 (8.7)	34 (11.6)	
Talc use				0.97
Never	221 (37.3)	111 (37.2)	110 (37.4)	
Ever	371 (62.7)	187 (62.8)	184 (62.6)	
Physical inactivity				0.88
Less than 2 h weekly	331 (61.5)	172 (61.2)	159 (61.9)	
2 + hours weekly	207 (38.5)	109 (38.8)	98 (38.1)	
Missing	54 (–)	17 (–)	37 (–)	
Hormonal- and reproductive-related factors				
PMH duration				0.58
None	488 (83.0)	250 (84.5)	238 (81.5)	
< 5 years	61 (10.4)	27 (9.1)	34 (11.6)	
5 + years	39 (6.6)	19 (6.4)	20 (6.8)	
Missing	4 (–)	2 (–)	2 (–)	
OC Duration				0.80
Never	178 (30.4)	86 (29.2)	92 (31.7)	
< 5 years	234 (40.0)	120 (40.7)	114 (39.3)	
5 + years	173 (29.6)	89 (30.2)	84 (29.0)	
Missing	7 (–)	3 (–)	4 (–)	
Parity				0.83
0	109 (18.4)	54 (18.1)	55 (18.7)	
1	105 (17.7)	48 (16.1)	57 (19.4)	
2	143 (24.2)	74 (24.8)	69 (23.5)	
3	113 (19.1)	57 (19.1)	56 (19.0)	
4+	122 (20.6)	65 (21.8)	57 (19.4)	
Other comorbidities				
Previous cancer diagnosis				
Prior cancer (excludes breast cancer)	27 (4.6)	11 (3.7)	16 (5.4)	0.30
Prior breast cancer*	39 (6.6)	25 (8.4)	14 (4.8)	0.08
Charlson comorbidity index^1^				0.30
0	216 (36.5)	113 (37.9)	103 (35.0)	
1	136 (23.0)	75 (25.2)	61 (20.7)	
2	99 (16.7)	44 (14.8)	55 (18.7)	
3+	141 (23.8)	66 (22.1)	75 (25.5)	
Clinical characteristics				
Time between baseline & follow-up (months)				
Median [Min, Max]		14.93 [6.18, 108.00]		
Mean (SD)		19.13 (14.48)		
Time between diagnosis & death or last contact (years)**				
Median [min, max]	4.81[0.45, 10.59]	6.21 [1.77, 10.59]	2.74 [0.45, 10.18]	
Mean (SD)	4.79 (2.62)	5.95 (2.33)	3.63 (2.38)	< 0.01
Vital Status as of 2021**				< 0.01
Alive	228 (38.5)	141 (47.3)	87 (29.6)	
Deceased	364 (61.5)	157 (52.7)	207 (70.4)	
Stage*				0.09
Localized	131 (23.8)	79 (27.5)	52 (19.7)	
Regional	52 (9.4)	27 (9.4)	25 (9.5)	
Distant	368 (66.8)	181 (63.1)	187 (70.8)	
Missing	41 (–)	11 (–)	30 (–)	
FIGO Stage				0.51
I	130 (25.6)	74 (27.4)	56 (23.5)	
II	57 (11.2)	31 (11.6)	26 (10.9)	
III	227 (44.7)	121 (44.8)	106 (44.5)	
IV	94 (18.5)	44 (16.3)	50 (21.0)	
Unknown/unstaged	84 (–)	28 (–)	56 (–)	
Histotype*				0.08
High-grade serous	397 (67.7)	204 (68.5)	193 (67.0)	
Low-grade serous	17 (2.9)	9 (3.0)	8 (2.8)	
Endometrioid	57 (9.7)	32 (10.7)	25 (8.7)	
Clear cell	23 (3.9)	17 (5.7)	6 (2.1)	
Mucinous^2^	29 (4.9)	13 (4.4)	16 (5.6)	
Carcinosarcoma	18 (3.1)	7 (2.4)	11 (3.8)	
Other epithelial	45 (7.7)	16 (5.4)	29 (10.1)	
Missing	6 (–)	0 (–)	6 (–)	
Debulking status, CA125*				0.07
Optimal	255 (66.6)	151 (72.6)	104 (63.8)	
Suboptimal	116 (30.3)	57 (27.4)	59 (36.2)	
No debulking surgery	12 (3.1)	1 (–)	11 (–)	
Missing	209 (–)	89 (–)	120 (–)	

*p < 0.10; **p < 0.05 when comparing follow-up to no follow-up

^1^Charlson index uses the following comorbidities: asthma, arthritis, diabetes, digestive issues, heart trouble, HIV/AIDs, kidney disease, liver disease, & stroke

^2^includes both invasive and borderline

Overall, almost a quarter of AACES participants had high Charlson scores (≥ 3 weighted score, 24%), more than half reported being obese (BMI one year prior to diagnosis ≥ 30 kg/m^2^, 59%), more than half reported never smoking (55%), almost two-thirds reported previous talc use (63%), almost two-thirds reported less than two h of physical activity per week (62%), the majority reported never using post-menopausal hormones (83%), and the majority reported ever using oral contraceptives (70%) (Table 3). Of note, obesity did not differ according to participation in the follow-up survey (Table 3). Those who did not participate in the follow-up survey were more likely to report they were a current smoker and less likely to have never smoked compared to those who participated in at least one follow-up survey (current: 37 vs 32%; never: 51 vs 59%) (Table 3). Participants who were deceased before follow-up were more likely to have higher scores on the Charlson comorbidity index than those who were lost to follow-up for other reasons, with 70 and 61% reporting a non-zero Charlson score, respectively (*p* = 0.086).

### Clinical, tumor, and genetic data

The distribution of the tumor characteristics including stage at diagnosis and histotype is shown in Table 3. Most participants were diagnosed at FIGO stage III/IV (63%) and most had high-grade serous tumors (68%). The least common histotypes were low-grade serous (3%) and carcinosarcoma (3%). Those who completed at least one follow-up survey were less likely to have stage III/IV disease compared to those who did not participate, 61 vs 66%, respectively. When comparing participants who completed at least one follow-up survey to those who did not complete a follow-up survey, participants were slightly more likely to be diagnosed with FIGO stage I disease, 27 vs 24%, respectively, and slightly less likely to be diagnosed with FIGO stage IV disease, 16 vs 21%, respectively. The distribution of histologic subtypes was similar across the baseline and follow-up groups, with most women having high-grade serous ovarian cancer.

Half of the women with high-grade serous ovarian cancer who were lost to follow-up were deceased at the first attempt of follow-up (50%), and this was the highest rate among the five major histotypes. Similarly, approximately half of the women diagnosed at FIGO stage III/IV were lost to follow-up because they were deceased at the first attempt to contact (51%). More than half of participants who had suboptimal debulking were lost to follow-up because they were deceased at the first attempted contact (56%).

The proportion of the five major histotypes among participants in AACES compared to the proportion in SEER data for Black women, respectively, are similar: 75.9 vs 77.8% high-grade serous ovarian, 3.3 vs 2.7% low-grade serous, 10.9 vs 7.4% endometrioid, 4.4 vs 4.6% clear cell, and 5.5 vs 7.5% mucinous (*p* > 0.10). Supplemental fig 1 shows Kaplan–Meier OS curves by the five major histotypes in AACES and in SEER. The poorest OS is seen for women with high-grade serous ovarian cancer, while women with endometrioid tumors have the best OS. To date in AACES, more than two-thirds of women who were diagnosed with high-grade serous ovarian cancer are deceased (69%), compared with approximately one-fifth of women diagnosed with endometrioid ovarian cancer (19%). When comparing these to the Kaplan–Meier estimates from SEER data, we see similar OS for women with high-grade serous, mucinous, and endometrioid cancers.

Twelve participants did not receive debulking surgery. Information on residual disease/debulking status was available on 340 participants (59%). Among women who were missing this information and whose CA125 level at the end of adjuvant chemotherapy was used as a proxy for debulking status (*n* = 43), 32 women were classified as having optimal debulking (CA125 < 35 units/mL) and 11 women were classified as having suboptimal debulking (CA125 ≥ 35 units/mL). By incorporating CA125 levels, information on debulking status was available on 383 participants (65%) and the missing data were reduced to 209 participants (35%). At baseline, approximately one-third of the participants had any residual disease, while one-third had no gross residual disease. Comparing participants by follow-up survey completion, women who completed at least one follow-up survey were more likely to have no residual disease. Optimal debulking status, which is correlated with residual disease, was also highest among those who participated in at least one follow-up survey.

No information on residual disease was recorded for approximately one-third of the participants. In Supplemental fig 3, we also show that the OS curve among those with missing debulking status fell in-between those whose debulking status was either optimal or suboptimal. Participants with optimal, suboptimal, and missing debulking status have a median OS of 7.1, 3.0, and 4.9 years, respectively. This suggests that data were missing at random with respect to debulking status and therefore imputation of the missing data would likely be unbiased. Using Cox proportional hazards analysis, we find a similar hazard ratio (HR) for the complete case group analysis (HR = 0.43; 95% CI = 0.32, 0.60) compared with the estimate computed using multiple imputation (HR = 0.46; 95% CI = 0.36, 0.60).

AACES has contributed significantly and will continue to contribute to the genetic susceptibility and tumor biomarker research of ovarian cancer in Black women. Our GWAS findings were reported by Manichaikul et al. [35] suggesting similarities and differences in genetic association in Black compared to White women. Initial results from multiplex immunofluorescence staining of immune markers and OS of Black women in AACES are found in Peres et al. [36] and show an attenuated inverse association with survival in Black compared to White women with EOC.

## Discussion

Here we describe a multi-level approach to determine associations between epithelial ovarian cancer survival and factors associated with the social and built environment, individual patient characteristics, and the tumor immune microenvironment with the goal to examine the full spectrum of exposure variables and clinical factors to help characterize the “whole person.” Our scientific rationale is based on the premise that multiple factors contribute to the poor survival of ovarian cancer among Black women compared with other racial and ethnic groups and that many of these factors have a synergistic relationship or mediate the relationship with inflammatory and immune processes. We will be using multi-level modeling to address different effects identified at different levels: individual, neighborhood, and census tract that will be used to formulate a general multi-level model for survival experience [37–39].

The purpose of the study is to generate needed evidence to address the racial disparity in EOC survival. Most large medical claims databases examining racial and ethnic differences in ovarian cancer survival lack information on lifestyle behaviors and beliefs that may be key to the interactions with the health care system. AACES provides a rigorously designed and truly unique resource to achieve our goal of understanding factors that influence mortality to better understand why Black women experience worse survival after a diagnosis of EOC. AACES integrates key data that will address this critical evidence gap and provide a major step forward in understanding multi-level predictors of poor EOC prognosis in Black women. The resulting evidence will inform translational strategies to reduce this racial disparity. To illustrate this point, we recently published a paper based on AACES phase 1 participants that showed perceived everyday discrimination was associated with prolonged symptom duration, whereas more commonly evaluated determinants of access to care and trust in physicians were not [40].

A major strength of AACES is that participants reside in over 12 geographic regions in the U.S., both in the east and west coast as well as southern and northern regions. With substantial proportions of the cohort having relatively low levels of household income, education, and insurance coverage, AACES will be well positioned to assess the contribution of social determinants of health to the racial disparity in ovarian cancer survival. Our biospecimen collection represents an unprecedented number of well-annotated specimens and an important resource for the molecular characterization of EOC diagnosed in Black women.

Related to social determinants of health, this cohort is also characterized by a high prevalence of obesity and comorbidities. A large study exploring racial and ethnic disparities found that 36% of Non-Hispanic White women are obese, while 59% of Non-Hispanic Black women are obese [41]. This prevalence of obesity in Black EOC cases is equivalent to the proportion found in our population. In another study using data from the National Health Interview Study (NHIS), the rates of multiple comorbidities are roughly similar to that in the AACES. When age adjusting their estimates, approximately 66% of Non-Hispanic Black women reported either having zero or one comorbidities, while approximately 74% of Non-Hispanic White women reported having 0 or 1 comorbidities [42]. While this raw count is not strictly comparable to the Charlson comorbidity index (some more severe comorbidities are weighted > 1), 60% of the women in our sample have a Charlson score of zero or one. This is naturally a slight undercount of number of comorbidities, so it makes sense for this proportion to fall under that of the general population.

The distribution of the variables related to social determinants of health along with the high prevalence of factors such as obesity and comorbidities that lead to worse prognosis will enable the AACES to generate needed evidence on the role that these factors, alone and in combination, that contribute to Black women having the lowest survival from ovarian cancer. Integrating information such as mentioned above related to perceived discrimination will further enrich the insights that can be gained in this patient population.

The distribution variables such as income, education, and insurance point to a generally lower SES. Having a lower SES may lead to diagnostic delay and less access to treatment after diagnosis, both of which can have impacts on cancer survival [43]. These effects can come from both the individual and the neighborhood level, so a multi-level approach that the wide variety of data collected within AACES will be key to fully evaluate these relationships.

Comparisons of the survival of women with EOC in phase 1 of AACES to that in the SEER database suggests that AACES phase 1 participants are representative of those who survive at least 10 months since their diagnosis. Therefore, our study population may underrepresent the sickest patients diagnosed with EOC. When conditioning on having survived at least 10 months, the survival among Black women in AACES is worse compared to White women diagnosed with EOC in the SEER database. Due to these findings, inferences from the AACES results will likely not be as generalizable to women with the most severe disease. The appearance of poorer low-grade serous ovarian cancer survival and better clear cell ovarian cancer survival in the AACES sample may be due to the small number of women with these histotypes in AACES. Some of this could also be due to differences in stage at diagnosis. Due to the underrepresentation of the sickest patients diagnosed with EOC, women diagnosed with clear cell tumors at a later stage and poorer prognosis could be missing from the AACES sample.

We compared baseline data among women in AACES phase 1 who did or did not participate in at least one follow-up survey showing differences with those completing at least one follow-up survey being younger at diagnosis, having fewer comorbid conditions, being less likely to have ever smoked, having less distant stage disease at diagnosis, and having less residual disease after debulking surgery. The higher proportion of women who completed a follow-up survey reporting a previous breast cancer could be underrepresenting women who had more severe breast cancer and did not survive long enough to develop ovarian cancer. Approximately 50% of study subjects completed at least one follow-up survey and these women had a median survival of more than 6 years compared to less than 5 years for AACES 1 phase participants overall. In SEER, the median survival time among Black women with EOC is approximately 5 years. Therefore, women completing the follow-up survey are survivors of a highly fatal disease and overrepresent younger women with less severe disease and lower comorbidity burden. Nevertheless, AACES appears to have a similar survival experience compared to the few studies addressing ovarian cancer survival. A study from Australia reported an average follow-up of 7.3 years with only 45% alive after 5 years from diagnosis [44] and a recent cohort study of ovarian cancer patients in China reported a median follow-up time of 3.1 years [45], although these studies do not include Black women with EOC.

We will continue to improve our methods for accrual of patients. As funding for AACES phase 2 began in 2020, we will continue adapting our methods to accommodate ongoing COVID 19 pandemic challenges. Our findings will have the potential to shed light on the causes of the persistent racial disparities in EOC survival and provide strategies toward cancer health equity.

## Supplementary Information

Below is the link to the electronic supplementary material.

Supplementary file1 (DOCX 418 KB)

## Data Availability

The datasets generated during and/or analyzed during the current study are available on reasonable request, in accordance with NIH data sharing policy.
