# Supplementary material for: Survival of epithelial ovarian cancer in Black women: a society to cell approach in the African American cancer epidemiology study (AACES)
Source: Cancer Causes Control. 2022 Dec 15;34(3):251–65. doi: 10.1007/s10552-022-01660-0 (PMC9753020; doi:10.1007/s10552-022-01660-0)
Supplement: Supplementary file 1 — Supplementary file1 (DOCX 418 KB) [file 10552_2022_1660_MOESM1_ESM.docx]

**Survival of epithelial ovarian cancer in Black women in the African American Cancer Epidemiology Study (AACES)**

Supplemental Figures and Tables

**Supplemental Figure 1**. Survival Curves by Major Histotypes in AACES and in SEER 2008-2013, Age-Standardized to AACES with ≥10 months survival

**
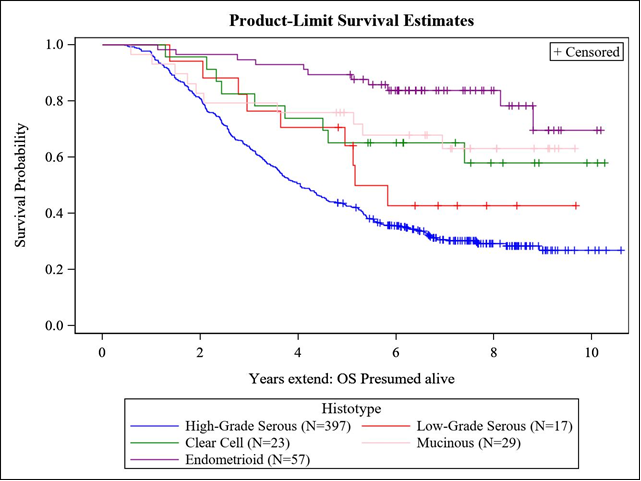

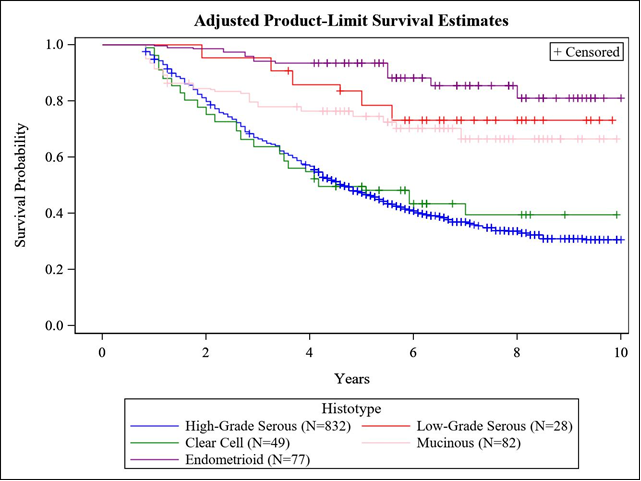
**

**Supplemental Figure 2**. Kaplan-Meier Curves by Debulking Status


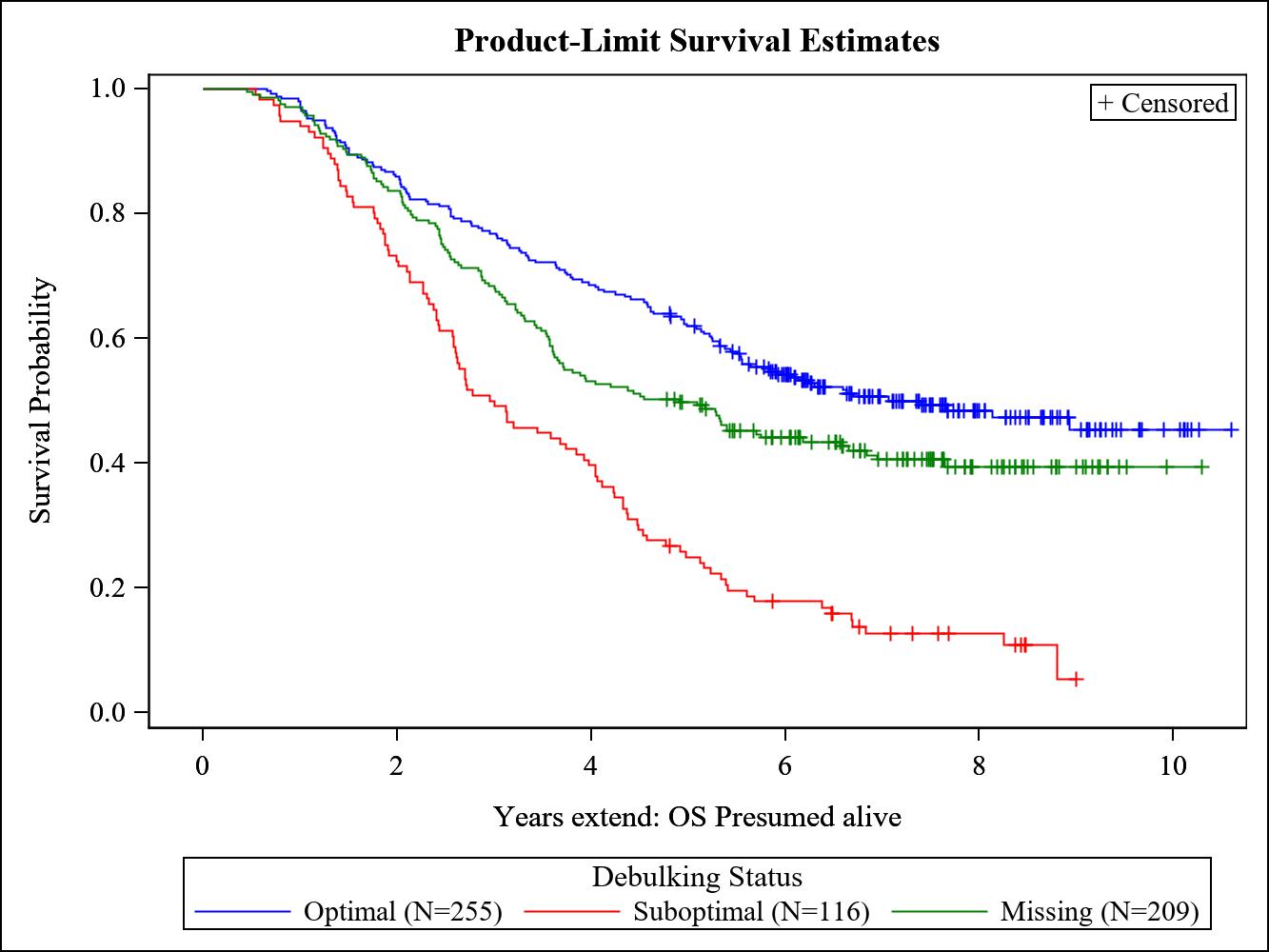


**Supplemental Figure 3**. Absolute Ovarian Cancer Incident Diagnoses for Black women by State, 2014-2018


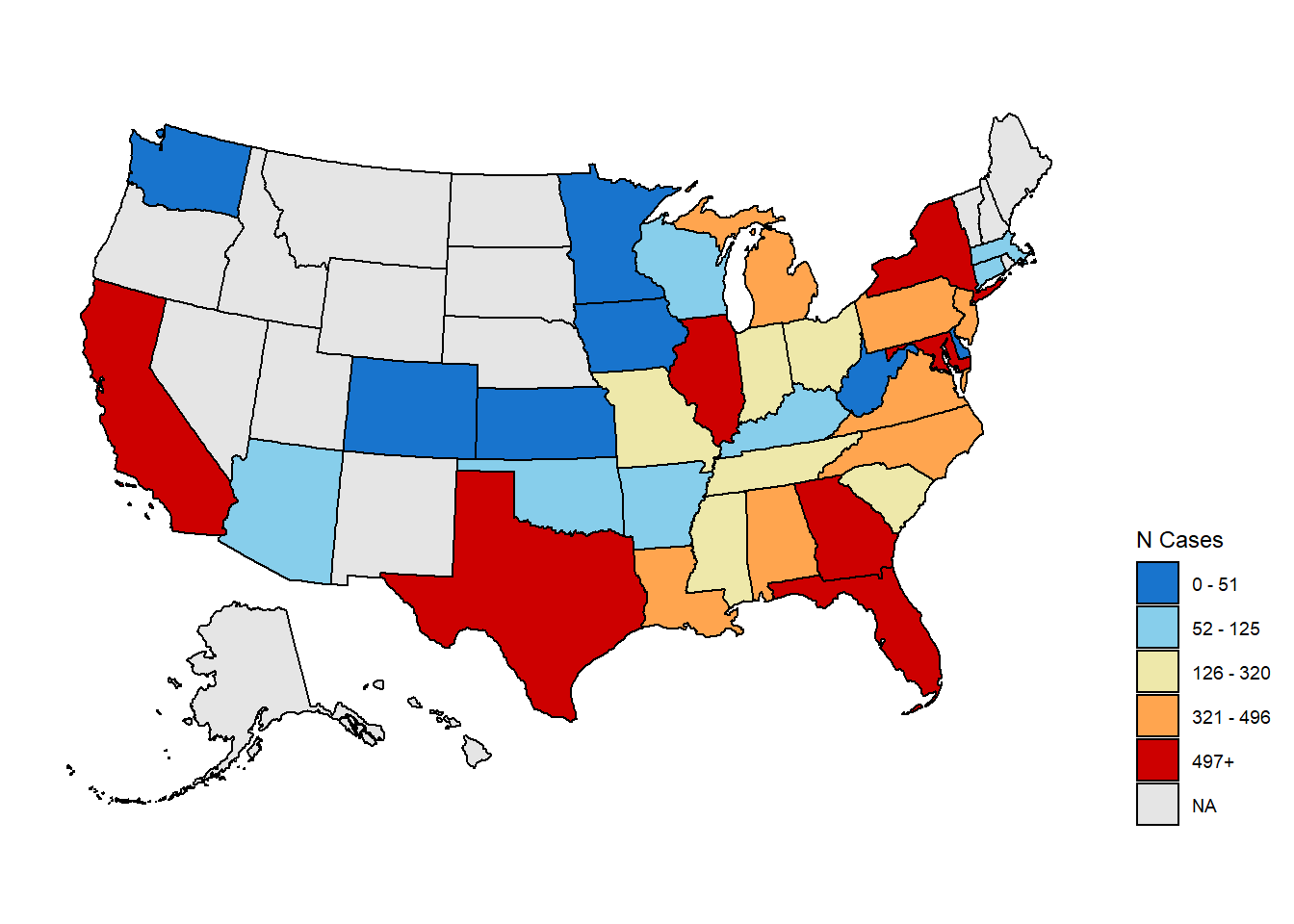


Data pulled from CDC United States Cancer Statistics: Data Visualizations

Star indicates site included in AACES Phase 1 or 2

**Supplemental Table 1.** Eligible ICD-0-3 Codes for AACES 2 Participants

| **ICD-0-3 Code** | **Description** |
| --- | --- |
| 8000/3 | Neoplasm, malignant |
| 8004/3 | Malignant tumor, spindle cell type |
| 8005/3 | Malignant tumor, clear cell type |
| 8010/3 | Carcinoma, NOS |
| 8011/3 | Epithelioma, malignant |
| 8020/3 | Carcinoma, undifferentiated, NOS |
| 8021/3 | Carcinoma, anaplastic, NOS |
| 8022/3 | Pleomorphic carcinoma |
| 8032/3 | Spindle cell carcinoma, NOS |
| 8046/3 | Non-small cell carcinoma |
| 8050/3 | Papillary carcinoma, NOS |
| 8120/3 | Transitional cell carcinoma, NOS |
| 8140/3 | Adenocarcinoma, NOS |
| 8144/3 | Adenocarcinoma, intestinal type |
| 8255/3 | Adenocarcinoma with mixed subtypes |
| 8260/3 | Papillary adenocarcinoma, NOS |
| 8290/3 | Oxyphilic adenocarcinoma |
| 8310/3 | Clear cell adenocarcinoma, NOS |
| 8313/3 | Clear cell adenocarcinofibroma |
| 8323/3 | Mixed cell adenocarcinoma |
| 8380/3 | Endometrioid carcinoma |
| 8381/3 | Endometrioid adenofribroma, malignant |
| 8382/3 | Endometrioid adenocarcinoma, secretory variant |
| 8383/3 | Endometrioid adenocarcinoma, ciliated cell variant |
| 8410/3 | Sebaceous adenocarcinoma |
| 8440/3 | Cystadenocarcinoma, NOS |
| 8441/3 | Serous tubalintraepithelial carcinoma |
| 8442/3 | Proliferating serous carcinoma, malignant |
| 8443/3 | Clear cell cystadenocarcinoma |
| 8444/3 | Clear cell cystic tumor, malignant |
| 8450/3 | Papillary cystadenocarcinoma, NOS |
| 8460/3 | Low-grade serous carcinoma |
| 8461/3 | High-grade serous carcinoma |
| 8462/3 | Papillary serous cystadenocarcinoma |
| 8470/3 | Mucinous cystadenocarcinoma, NOS |
| 8471/3 | Papillary mucinous cystadenocarcinoma |
| 8472/3 | Mucinous cystadenocarcinoma |
| 8474/3 | Seromucinous carcinoma |
| 8480/3 | Mucinous adenocarcinoma |
| 8481/3 | Mucin-producing adenocarcinoma |
| 8482/3 | Mucinous adenocarcinoma, endocervical type |
| 8560/3 | Adenosquamous carcinoma |
| 8570/3 | Adenocarcinoma with squamous metaplasia |
| 8575/3 | Metaplastic carcinoma, NOS |
| 8950/3 | Mullerian mixed tumor |
| 8951/3 | Mesodermal mixed tumor |
| 8980/3 | Carcinosarcoma, NOS |
| 9000/3 | Brenner tumor, malignant |
| 9014/3 | Serous adenocarcinofibroma |
| 9015/3 | Mucinous adenocarcinofibroma |
| 9110/3 | Mesonephric adenocarcinoma |
| 9111/3 | Mesonephric-like adenocarcinoma |
